# Enhanced Biodegradation Rate of Poly(butylene adipate-co-terephthalate) Composites Using Reed Fiber

**DOI:** 10.3390/polym16030411

**Published:** 2024-02-01

**Authors:** Jia Xu, Kunpeng Feng, Yuan Li, Jixing Xie, Yingsai Wang, Zhiqiang Zhang, Qing Hu

**Affiliations:** 1 Xiong’an Institute of Innovation, Baoding 071700, China; xujia@xii.ac.cn (J.X.);; 2 College of Chemistry and Environment Science, Hebei University, Baoding 071000, China

**Keywords:** poly(butylene adipate-co-terephthalate), reed fibers, biodegradation, enzyme degradation, compost

## Abstract

To enhance the degradability of poly(butylene adipate-co-terephthalate) (PBAT), reed fiber (RF) was blended with PBAT to create composite materials. In this study, a fifteen day degradation experiment was conducted using four different enzyme solutions containing lipase, cellulase, Proteinase K, and esterase, respectively. The degradation process of the sample films was analyzed using an analytical balance, Fourier transform infrared spectroscopy (FTIR), scanning electron microscopy (SEM), and differential scanning calorimetry (DSC). The PBAT/RF composites exhibited an increased surface hydrophilicity, which enhanced their degradation capacity. Among all the enzymes tested, lipase had the most significant impact on the degradation rate. The weight loss of PBAT and PBAT/RF, caused by lipase, was approximately 5.63% and 8.17%, respectively. DSC analysis revealed an increase in the melting temperature and crystallinity over time, especially in the film containing reed fibers. FTIR results indicated a significant weakening of the ester bond peak in the samples. Moreover, this article describes a biodegradation study conducted for three months under controlled composting conditions of PBAT and PBAT/RF samples. The results showed that PBAT/RF degraded more easily in compost as compared to PBAT. The lag phase of PBAT/RF was observed to decrease by 23.8%, while the biodegradation rate exhibited an increase of 11.8% over a period of 91 days. SEM analysis demonstrated the formation of more cracks and pores on the surface of PBAT/RF composites during the degradation process. This leads to an increased contact area between the composites and microorganisms, thereby accelerating the degradation of PBAT/RF. This research is significant for preparing highly degradable PBAT composites and improving the application prospects of biodegradable green materials. PBAT/RF composites are devoted to replacing petroleum-based polymer materials with sustainable, natural materials in advanced applications such as constructional design, biomedical application, and eco-environmental packaging.

## 1. Introduction

Plastics have excellent properties, such as easy processing, high strength, corrosion resistance, and affordability, making them indispensable materials in daily life, production, and processing. Their products have penetrated various fields of social life, including disposable daily necessities, agriculture, industry, and biomedicine. These products have significantly transformed human life [1,2]. However, traditional plastics are difficult to degrade under natural conditions. Since 1950, humans have produced a total of about 9 billion tons of plastic, of which more than 90% has been landfilled, abandoned, or incinerated. According to the current production rate, it is estimated that 12 billion tons of plastic will be discarded by 2050, resulting in an irreversible impact on global plastic pollution [3]. In recent years, there has been a rapid development in the research of biodegradable materials, leading to increased attention on biodegradable plastics due to their excellent biodegradability. They have already proven their value in many fields and have become one of the most effective ways to reduce “white pollution”, protect ecological balance, and achieve sustainable development.

PBAT is an aliphatic aromatic biodegradable polyester, which is considered one of the most promising green polymer materials for replacing traditional plastics. It possesses excellent flexibility, ductility, heat resistance, impact resistance, and biocompatibility [4]. The biodegradation behavior of PBAT is primarily attributed to microbial degradation and hydrolysis. When composted or buried in the soil, enzymatic hydrolysis is the primary method of degradation of polyester. The ester bonds within the polyester backbone are cleaved by specific enzymes, including lipase, esterase, Proteinase, and keratinase, which are produced by microorganisms. This process generates oligomers and monomers that can be further utilized by microorganisms [5]. Current research shows that the degradation of PBAT in practical application processes is not optimal. Wu et al. studied the degradation characteristics of PBAT film in cotton fields. After 180 days, only small cracks were observed on the surface, and the degree of degradation was not significant [6]. The melting point and mechanical properties of PBAT are similar to those of low-density polyethylene, making it suitable for film production [7]. This is particularly advantageous given the increasing market demand for such films. PBAT has become the most widely used biodegradable material in the packaging, agriculture, and transportation industries. However, its high production cost, poor rigidity, and long degradation cycle have limited its popularity and application. Therefore, improving the degradation rate of PBAT under natural and composting conditions has become an urgent issue.

Plant fiber has the characteristics of high specific strength and modulus, strong processability, widespread availability, renewability, and full biodegradability. It is the most abundant natural polymer material in nature. Blending and modifying it with biodegradable polyester is an important method for effectively regulating the mechanical properties, degradation properties, and production costs of its composites [8]. Many different natural plant fibers, such as miscanthus fiber, jute fibers, hemp fibers, coconut, coffee grounds, and wheat straw, have been used by numerous researchers to prepare PBAT biodegradable composites [9,10,11,12,13,14,15]. The composite materials, which are made by blending PBAT with plant fibers, can be degraded by microorganisms. The degradation process results in the production of small molecular substances, such as carbon dioxide and water. Lamsaf et al. discovered that the incorporation of hemp fiber into PBAT greatly improved the compostability of PBAT. The composting rate of the hemp fiber/PBAT blend was found to be twice as fast as that of neat PBAT [16]. Additionally, Zeng et al. confirmed the favorable biodegradability of hemp fiber/PBAT composites through soil burial degradation tests. Since most natural plant fibers have high water absorption and wettability, they can promote the degradation of composites [17]. As a result, the degradation rate of PBAT composites can be significantly accelerated. Giri et al. found that as the fiber content of wheat straw in the composites with PBAT increased, their hydrophilicity also increased, thereby enhancing the biodegradability of the composites [18].

Reed (*Phragmites australis*) is a perennial herbaceous plant that is widely distributed around the world. It is characterized by its adaptability, rapid growth, high yield, and ability to thrive without the need for fertilization. There are currently 14 major reed-producing areas in China, covering an area of more than 1.3 million hm^2^, with an annual production of more than 2.2 million tons [19]. Reed fiber has several advantages, including low density, affordability, environmental friendliness, and excellent mechanical properties. As a result, it is considered a highly promising natural plant fiber source. Using reed fiber to prepare biodegradable composites can play a role in improving performance, reducing costs, increasing degradation rates, and reducing the negative impacts of the decay and decomposition of waste biomass on the environment. For example, Wang et al. obtained polylactic acid/reed straw nanofiber composites with a higher tensile strength, fracture elongation, and heat deflection temperature compared to neat polylactic acid (PLA) [20]. The reed/PLA composites, which exhibited a 39% increase in tensile modulus and a 45% increase in flexural modulus compared to neat PLA, were prepared by Fiore et al. [21]. Additionally, Suárez et al. conducted simulated composting tests at room temperature. The weight loss of PLA composites containing 10% reed fibers during the test cycle was 4.6 times greater than that of neat PLA [22]. Xie et al. investigated the effects of preparing large-grained reed fillers on the degradation properties of PBAT composites [23]. During the enzymatic degradation process by lipase, composites showed a more significant decrease in quality compared to neat PBAT. With the continuous development of PBAT-based composites for large-scale applications, studying the degradation properties of these composites is particularly important.

In recent years, there has been extensive research in the literature on the use of natural plant fibers to obtain biodegradable composites. However, this research has mainly focused on composite preparation methods, interface modification, and mechanical properties [24,25]. There is a scarcity of research on the multiscale biodegradation of PBAT-based composites. Most applications require careful consideration of the degradation properties of biodegradable polyesters in order to meet both the performance requirements during use and the degradation requirements after disposal. The objective of this study was to investigate the enzymatic degradation of PBAT/RF composites and their biodegradation behavior under controlled composting conditions. Additionally, the study aimed to characterize the structural changes of the composites using various tests in order to assess the potential impact of reed fibers on the biodegradation rate of PBAT composites used for film-based applications. In this study, composites were prepared using microfibrillated reed fibers and PBAT, to enhance the degradation rate of PBAT. We conducted a quantitative evaluation of the degradation behavior of PBAT/RF composites to understand the degradation mechanism. We also studied the influence of reed fiber on the abiotic and biotic degradation of PBAT, offering a point of reference for improving the degradation process. Furthermore, the results of this study will provide a theoretical reference for the design, modification, and application of PBAT composites that are more conducive to degradation. This will expand their application areas and provide higher economic benefits for industrial production.

## 2. Materials and Methods

### 2.1. Materials

PBAT (TH801T) was purchased from Xinjiang Blue Ridge Tunhe Polyester Co., Ltd (Changji, China). It has a specific gravity of 1.21 g/cm^3^ and a melt mass flow rate (MFR) of 5.0 g/10 min (190 °C), with a number-average molecular weight of 6.12 × 10^4^ g/mol. Reeds were collected from the shore of the lake (Baiyangdian, Baoding, China). For enzymatic degradation, the following enzymes and reagents were used: Proteinase K (≥30 units/mg) from *Tritirachium album* (Blirt, Gdansk, Poland), esterase (≥10 units/mg) from *Bacillus subtilis* (Sigma-Aldrich, Bavaria, Germany), lipase (15–25 units/mg) from *Candida rugosa* (Sigma-Aldrich, Bavaria, Germany), and cellulase (≥200 FPU/mL) from *Trichoderma reesei* (Novozymes A/S, Copenhagen, Denmark). Tris(hydroxymethyl)aminomethane-HCl (0.1 M, pH = 8.0, Sigma-Aldrich, Germany) and citric acid-sodium citrate (0.1 M, pH = 4.8, Sigma-Aldrich, Germany) were used as buffers for enzyme degradation, sodium azide (NaN_3_) (Sigma Chemical Co., St. Louis, MI, USA) was used as an antifungal agent, and calcium chloride (Sigma Chemical Co., USA) was used as an activator of enzyme degradation. Other chemical reagents were obtained from Sinopharm Chemical Reagent (Shanghai, China).

### 2.2. Preparation of Sample Films

The reed fibers were dried and ground, and the resulting fibers were passed through 500-mesh sieves. PBAT granules and RF were dried at 60 °C for 24 h before extrusion to eliminate moisture. PBAT and RF were weighed in a ratio of 8:2, then the materials were evenly shaken for 20 min by a mixer (SHR-10, Zhangjiagang Blue Sky Machinery Co., Ltd., Zhangjiagang, China). The blend of PBAT and RF was compounded using a twin-screw extruder (BD-8859-A, Dongguan Baoding Precision Instrument Co., Ltd., Dongguan, China) at a speed of 40 rpm and a temperature range of 140–150 °C. The feed rate into the extruder is 1.0 kg/h. The extruder had the following characteristic features: a screw diameter of 20 mm and an L/D ratio of 40. The screw is a co-rotating meshing type, and the threaded part consists of several different forms of threaded units that assemble like building blocks. For comparison, neat PBAT was extruded and granulated to produce pellets under identical conditions. The extruded pellets were dried in an oven at 60 °C for 24 h [26]. PBAT and PBAT/RF films were prepared by extrusion blowing blends using a single screw extruder (SCM25, Zhangjiagang City Lianjiang Machinery Co., Ltd., Zhangjiagang, China) at a speed of 300 rpm and a temperature of 150 °C. The films will be referred to as PBAT and PBAT/RF, and the thickness of the resulting films was 20–25 μm. The film thickness is measured using the detection method specified in GB/T 6672-2001, using a thickness tester (PY-H606E, Shenzhen Puyun Electronic Co., Ltd., Shenzhen, China) [27].

### 2.3. Enzymatic Degradation Tests

The enzymatic degradation of PBAT and PBAT/RF was evaluated using Proteinase K, esterase, lipase, and cellulase. The sample films were cut with dimensions of 20 mm × 20 mm × 0.035 mm. These films were dried in a vacuum oven at 50 °C for 24 h and weighed before being immersed in the degradation medium. The samples for Proteinase K, esterase, and lipase degradation were placed into vials containing 20 mL of Tris-HCl buffer solution and 8 mg of enzymes. The samples for cellulase degradation were immersed in 20 mL of citric acid-sodium citrate buffer solution and 4 mL of enzymes. Sodium azide (2 mg) and calcium chloride (1 mg) were added to all treated solutions. Enzymatic degradation was performed in an incubator at 50 °C, and the buffer–enzyme system was renewed every 24 h to maintain the enzymatic activity [28]. The samples (in triplicate) were taken out at 3, 6, 9, 12, and 15 days. They were washed with water, wiped, and weighed. They were completely dried in a vacuum oven at 50 °C for 24 h, weighed, and then returned to the same enzyme solution. The percentage of mass increase due to water absorption (*W*_*a*_) was calculated using Equation (1) [29], and the percentage of weight loss (*W*_*b*_) was calculated according to Equation (2):

(1) $$W_{a}\left(\%\right)=\frac{w_{0}-w_{1}}{w_{0}}\times 100$$

where *w*_0_ is the weight of sample removed from enzyme solution, *w*_1_ is the weight of completely dry sample after enzymatic degradation.

(2) $$W_{b}\left(\%\right)=\frac{w_{2}-w_{3}}{w_{2}}\times 100$$

where *w*_2_ is the dry weight of sample before enzymatic degradation, *w*_3_ is the dry weight of sample after enzymatic degradation.

### 2.4. Composting Degradation Tests

For composting biodegradation, mature compost was obtained from the Beijing Nangong composting plant. The physical and chemical properties of the compost are analyzed in Table 1. Four groups of composting reactors were utilized, and tests for each composition were conducted in triplicate. The reactors were placed in a lightless incubator at a temperature of 58 ± 2 °C and a humidity level of 50–55% for 90 days [30]. The humidity, mixing, and aeration in all the reactors were controlled according to the requirements established by GB/T19277-2011 [31]. Compost humidity was adjusted every three days, and the samples were stirred to prevent clumping. In the first group, the vessels contained 600 g of compost as a blank control. In the second group, the vessels contained a mixture of 600 g of compost (dry basis) and 100 g of powdered cellulose (20 μm, Sigma-Aldrich, Germany) as the control sample (a). In the third group, the vessels contained a mixture of 600 g of compost (dry basis) and 100 g of reed fibers (same as in PBAT/RF composites) as the control sample (b). In the fourth group, the vessels contained 600 g of compost and 100 g of neat PBAT. In the fifth group, the vessels contained 600 g of compost and 100 g of PBAT/RF. The sample films were cut with dimensions of 5 mm × 5 mm × 0.035 mm. The CO_2_ that was released during the composting biodegradation process was captured in 1M NaOH solutions. At intervals of seven days, the NaOH solution was withdrawn and analyzed by titration with a standard HCl solution to determine the amount of CO_2_ evolved. After titration, a fresh 1 M NaOH solution was added to the vessels. The total CO_2_ evolved during the test was calculated with reference to the control vessel. The experimental results of each sample are the average values obtained from three samples. The rate of biodegradation (*D*_*t*_) was evaluated by using Equation (3) [32]:

(3) $$D_{t}(\%)=\frac{{\left(CO_{2}\right)}_{T}-{\left(CO_{2}\right)}_{B}}{{Th}_{co_{2}}}\times 100$$

where (*CO*_2_)_*T*_ represents the amount of CO_2_ produced in the sample, (*CO*_2_)_*B*_ represents the amount of CO_2_ produced in the blank control, and *Th*_*CO*2_ represents the theoretical amount of CO_2_ that the sample films can produce in each composting reactor. It is determined using a total organic carbon analyzer (TOC, TOC-2000, Shanghai Metash Instruments Co., Ltd., Shanghai, China), and then calculated.

### 2.5. Characterization

#### 2.5.1. Scanning Electron Microscopy

After the enzymatic degradation and composting biodegradation processes, the surface morphology of the sample films was observed using a scanning electron microscope (SEM, TM4000PLUS, Hitachi, Tokyo, Japan) operated at 15 kV. The samples were sputter-coated with gold prior to observation.

#### 2.5.2. Differential Scanning Calorimetry

Differential Scanning Calorimetry (DSC, DSC 3+, Mettler-Toledo, Zurich, Switzerland) was used to determine the melting behavior and crystallinity of the film samples before and after enzymatic degradation. The sample (5–10 mg) was placed into a hermetically sealed aluminum pan. The sample and reference pans were kept in a nitrogen atmosphere with a flow rate of 50 mL/min. The specimens were heated at a rate of 10 °C/min from −20 °C to 160 °C and held isothermally at 160 °C for 5 min to erase the thermal history. Then, they were cooled from −20 °C at 10 °C/min. Finally, in the second heating run, they were heated from −20 °C to 160 °C at the same heating rate [33]. The melting temperature (*T*_*m*_) and heat of fusion (Δ*H*_*m*_) were analyzed based on the data obtained during the second heating. The degree of crystallinity (*X*_*c*_) was calculated using Equation (4):

(4) $$X_{c}(\%)=\frac{{\Delta H}_{m}}{W_{P}\times {\Delta H}_{m}^{0}}\times 100$$

where Δ*H*_*m*_ is the measured heat of fusion of the sample, *W*_*p*_ is the weight fraction of the PLA phase in the composite, Δ$H_{m}^{0}$ Is the melting enthalpy of 100% crystalline PBAT, which is calculated as 114 J/g [34].

#### 2.5.3. Fourier Transform Infrared Spectrometry

Before and after enzymatic degradation, the samples were tested using a Fourier transform infrared spectrometer (FTIR, Spectrum 3, Perkin Elmer, Shelton, CT, USA). The samples were analyzed using the attenuated total reflection (ATR) mode. The spectra were recorded with 16 scans at a resolution of 4 cm^−1^, covering wavelengths from 4000 to 500 cm^−1^.

## 3. Results and Discussion

### 3.1. Enzymatic Degradation

#### 3.1.1. Weight Loss Analysis

**Figure 1:**
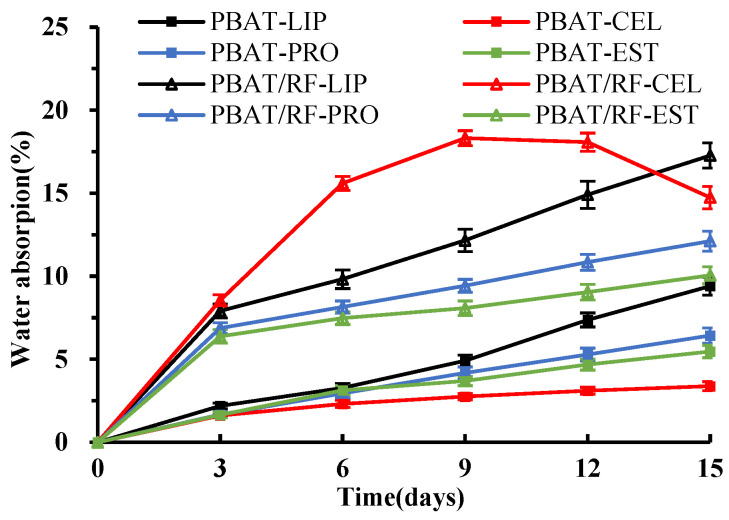
The water absorption of PBAT and PBAT/RF composites was evaluated over different durations of enzymatic degradation (LIP: lipase, CEL: cellulase, PRO: Proteinase K, EST: esterase).

The water absorption of the material is an important factor that affects the degradation rate. PBAT is a typically hydrophobic polyester and has poor water absorption. The reed fiber has a high cellulose content, which contains many hydroxyl groups and has a porous tubular structure. This structure promotes the adsorption and penetration of water molecules and enzyme proteins. Figure 1 illustrates the water absorption of the sample during the degradation process catalyzed by various enzymes. The overall water absorption rate of PBAT/RF composites was higher than that of neat PBAT. This indicates that the addition of reed fiber effectively improved the wettability of the material, resulting in the faster adhesion of the enzyme solution to the surface of the materials and facilitating its penetration into the interior. During the degradation period of 0 to 9 days, the water absorption of PBAT in all enzyme solutions exhibited a gradual increase. However, the water absorption of PBAT/RF composites was notably higher than that of PBAT during the initial measurement and continued to increase as the degradation time progressed. During the degradation period of 9 to 15 days, the water absorption of PBAT in different enzyme solutions appeared to vary. Lipase, Proteinase K, and esterase exhibited a significantly faster rate of water absorption, particularly for the lipase degradation reaction. After 15 days, the water absorption rate reached 9.41%. Lipase efficiently catalyzes the hydrolysis of the ester bond in the molecular chain of PBAT, leading to an increase in hydroxyl and carboxyl groups. This enhances the hydrophilicity of PBAT and intensifies the adsorption of water molecules. The enzymatic hydrolysis of PBAT results in the erosion and porosity of the surface, which causes water molecules to accumulate in the interstitial space and pores, thereby increasing the material’s water adsorption. Samples of cellulase exhibited a similar water absorption trend as in the previous stage, with the water absorption rate of PBAT/RF composites reaching only 3.38% at the end of the reaction. The water absorption rate of PBAT/RF composites showed a leveling off in lipase, Proteinase K, and esterase, and a decrease from 18.32% to 14.74% in cellulase.

**Figure 2:**
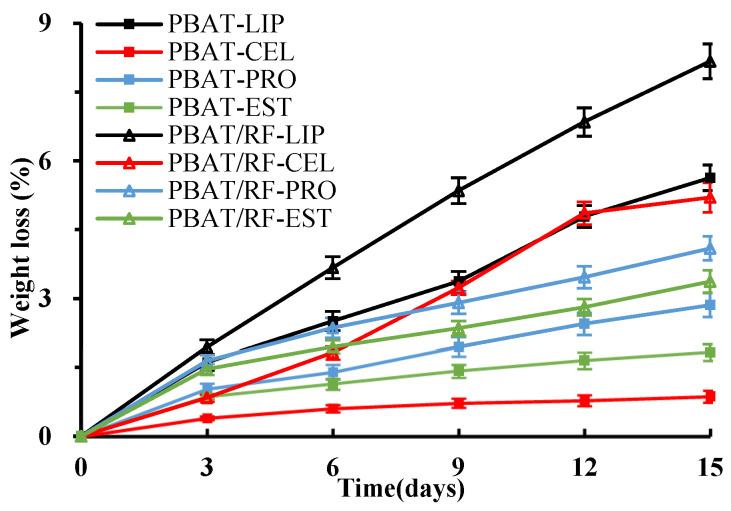
The weight loss of PBAT and PBAT/RF composites was measured over different durations of enzymatic degradation (LIP: lipase, CEL: cellulase, PRO: Proteinase K, EST: esterase).

Figure 2 illustrates the weight loss of the sample during degradation by various enzyme solutions. It demonstrates a correlation with the water absorption rate, indicating that the weight loss of PBAT/RF composites was higher than that of PBAT in the same enzymatic degradation reaction. Furthermore, the weight loss increased gradually over time. Lipase had the greatest influence on the degradation rate of the two types of samples. The weight loss of PBAT and PBAT/RF composites was 5.63% and 8.17% after 15 days, respectively. However, cellulase had the least impact on the degradation of PBAT. The weight loss was only 0.87% after 15 days. PBAT/RF composites exhibited an inconsistent performance in cellulase, and the weight loss of the samples demonstrated a pattern of rapid increase followed by a gradual stabilization over time. The results showed that the addition of reed fiber to PBAT improved the degradation performance of the composites. The reed fiber in the composite serves as a transport medium for the solution, increasing the surface area of PBAT in contact with the enzyme. This allows the solution to penetrate from the surface of the samples into the interior of the PBAT matrix [35]. Whereas, the differences in the rate of weight loss of the samples were related to the properties of the enzymes used, including reaction conditions, activity, and stability. Lipase, Proteinase K, and esterase all belong to the serine hydrolase family, which can catalyze the hydrolysis of ester bonds and contribute to the degradation of polymers with a polyester structure [36,37]. Cellulase is a specific type of glycoside hydrolase that catalyzes the hydrolysis of the β-1,4-glycosidic bonds in cellulose. The weight loss of the PBAT/RF composites in cellulase solution mainly originates from the degradation of reed fiber, which leads to a decrease in their water absorption.

#### 3.1.2. SEM Morphology

**Figure 3:**
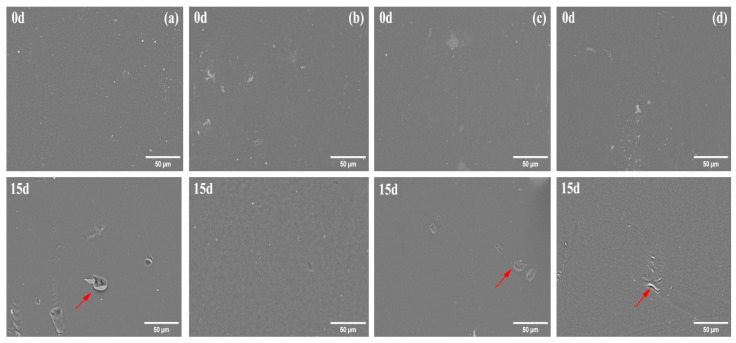
SEM micrographs of PBAT under various enzymatic treatments before and after degradation for 15 days (**a**) Lipase, (**b**) Cellulase, (**c**) Proteinase K, (**d**) Esterase. (Red arrows indicate cracks, pores, and corrosion areas that form on the surface of PBAT after enzymatic degradation).

**Figure 4:**
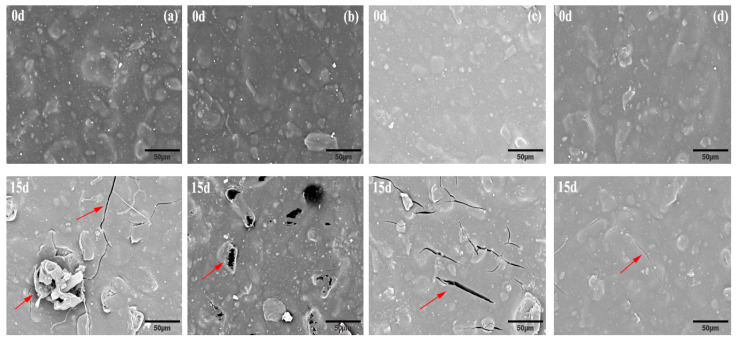
SEM micrographs of PBAT/RF composites under various enzymatic treatments before and after degradation for 15 days (**a**) Lipase, (**b**) Cellulase, (**c**) Proteinase K, (**d**) Esterase. (Red arrows indicate cracks, pores, and fibers that form on the surface of PBAT/RF composites after enzymatic degradation.)

The surface micromorphology changes of PBAT and PBAT/RF composites before and after enzymatic degradation were observed using SEM, as depicted in Figure 3 and Figure 4. The surface of PBAT samples was relatively smooth before degradation. However, after 15 days of treatment with lipase, Proteinase K, and esterase, the surface of PBAT exhibited unevenness, cracks, pores, and corrosion areas. This was particularly evident in the samples treated with lipase solution. The diameter of the surface pores and corrosion areas after degradation was more significant in this sample compared to the others. This suggests that the degree of degradation of PBAT by lipase was greater than that of Proteinase K and esterase. After 15 days of cellulase treatment, the surface roughness of PBAT increased, but there was no obvious erosion phenomenon. This increase in roughness can be attributed to the hydrolytic degradation of PBAT by the cellulase solution, which only occurs on the surface. The surface of the PBAT/RF composites was generally intact and protruding prior to degradation. After degradation, it is evident that significant cracks and pores have formed around the reed fibers. Additionally, the texture of the fibers was visible in specific areas. In the cellulase-treated samples, the surface of the composites underwent the most significant changes. The structure of the reed fibers was destroyed, leading to partial dissociation and the formation of numerous pores. On the other hand, in the presence of lipase, the PBAT/RF composites exhibited more noticeable erosion on the surface, with the fibers becoming exposed from the encapsulation of the PBAT matrix. Stepczyńska et al. observed similar findings in their research on the enzymatic degradation of PLA composites with flax fiber [38]. The analysis of the sample surface confirmed the weight loss measurements of enzymatic degradation of the samples. The PBAT/RF composites were found to be more hydrophilic than neat PBAT. This increased hydrophilicity allowed the enzyme solution to easily penetrate the reed fibers, facilitating the enzymatic hydrolysis reaction and accelerating the rate of degradation.

#### 3.1.3. DSC Analysis

**Figure 5:**
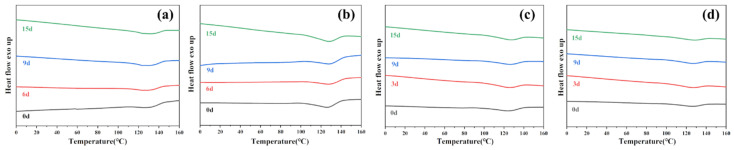
DSC thermograms from the second heating of PBAT under various enzymatic treatments before and after degradation for 3 days, 9 days, and 15 days (**a**) Lipase, (**b**) Cellulase, (**c**) Proteinase K, (**d**) Esterase.

**Figure 6:**
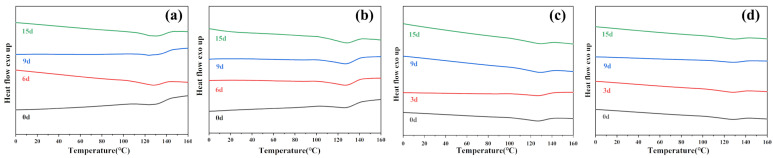
DSC thermograms from the second heating of PBAT/RF composites under various enzymatic treatments before and after degradation for 3 days, 9 days, and 15 days (**a**) Lipase, (**b**) Cellulase, (**c**) Proteinase K, (**d**) Esterase.

Figure 5 and Figure 6 show the impact of enzymatic degradation on the thermal properties of PBAT and PBAT/RF composites, respectively. The melting temperature (*T*_*m*_) of the sample increased with the degradation time when exposed to lipase, Proteinase K, esterase, and cellulase. PBAT is a semi-crystalline polymer consisting of polybutylene terephthalate (PBT) and polybutylene adipate (PBA). The melting points of PBT and PBA are 225 °C and 60 °C, respectively. Degradation priority occurs preferentially in the fragile aliphatic butylene adipate units (BA), and the relative content of butylene terephthalate units (BT) in the films increases after degradation, resulting in an increase in the melting temperature during degradation [34]. After 15 days of enzymatic degradation of both samples, the sample where PBAT/RF was degraded in the presence of lipase showed the most significant change in Δ*T*_*m*_, with an increase of 3.1 °C. On the other hand, the sample where PBAT was degraded in the presence of cellulase had the least change, with a Δ*T*_*m*_ of only 0.6 °C.

As can be seen in Table 2, all samples showed an increase in crystallinity after enzymatic degradation. This indicates that amorphous regions are more susceptible to degradation during enzymatic hydrolysis, resulting in a better crystalline structure with fewer defects [39]. The samples of PBAT/RF composites subjected to degradation in four enzyme solutions showed a greater crystallization ability compared to PBAT. The presence of reed fibers increased the hydrophilicity of the material and promoted the degradation of the PBAT matrix by the enzyme solution. This led to the breaking of the chains of the macromolecules in amorphous regions and the shortening of the chains of the macromolecules. As a result, the material gained higher mobility and malleability, and an improved crystallinity.

#### 3.1.4. FTIR Analysis

**Figure 7:**
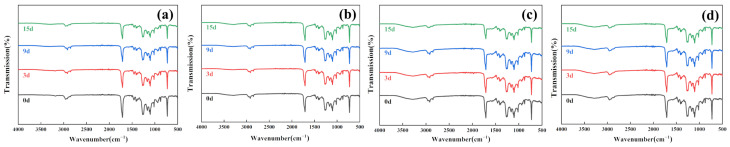
FTIR spectra of PBAT under various enzymatic treatments before and after degradation for 3, 9, and 15 days (**a**) Lipase, (**b**) Cellulase, (**c**) Proteinase K, (**d**) Esterase.

**Figure 8:**
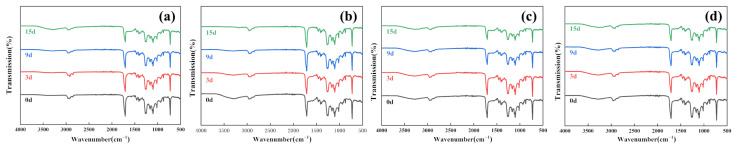
FTIR spectra of PBAT/RF composites under various enzymatic treatments before and after degradation for 3, 9, and 15 days (**a**) Lipase, (**b**) Cellulase, (**c**) Proteinase K, (**d**) Esterase.

The changes in the FTIR spectra of PBAT and PBAT/RF composites during enzymatic degradation are illustrated in Figure 7 and Figure 8. Following the incorporation of reed fibers into PBAT, the PBAT matrix enveloped the fibers, resulting in the overlapping of the peaks of reed fibers with those of PBAT. As a result, there was no discernible difference in the characteristic absorption peaks of the two samples prior to degradation. After treating the samples with lipase, Proteinase K, and esterase, there were no apparent changes in the position of the main characteristic absorption peaks in the FTIR spectra. However, a decrease in the intensity of the C–H asymmetric and symmetric telescopic vibrational absorption peaks at 2957 cm^−1^ and 2850 cm^−1^ was observed. Furthermore, the absorption intensity of the –C=O characteristic peak at 1712 cm^−1^ gradually weakened, which proved that the ester bond in the main chain was broken under the catalytic action of the enzyme. This characteristic is more evident in the PBAT/RF composite samples. However, cellulase does not exhibit the specific catalytic degradation of PBAT. The spectra of the samples before and after degradation show minimal changes in the aforementioned areas [40].

### 3.2. Composting Biodegradation

**Figure 9:**
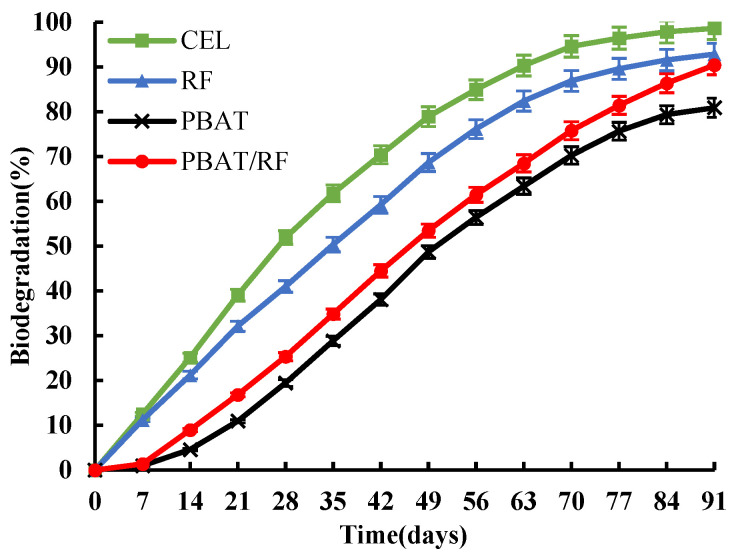
The biodegradation curves for cellulose, reed fiber, PBAT, and PBAT/RF composites under industrial composting conditions.

The release of CO_2_ over time during biodegradation was monitored for all samples under controlled composting conditions, as depicted in Figure 9. The test determined that the blank compost produced an average of 69.1 mg of CO_2_ per g of volatile solids in the first 10 days. This indicates that the mature compost used was rich in active microorganisms and met the requirements of the relevant standards. In the biodegradation curve, cellulose (positive control a) and reed fiber (positive control b) did not exhibit a lag phase. The biodegradation rate increased rapidly as time extended from 0 to 70 days and reached a stable stage after 70 days. The biodegradation rates at the end of the test reached 98.6% and 92.7%, respectively. Cellulose was degraded by 70% within the first 42 days, confirming the effectiveness of the test system. The study reports that natural plant fibers can decompose by more than 90% in 6 months, and reed fibers demonstrated good degradability in the tests. The reason why the decomposition rate of lignin is lower than that of cellulose within the same time is mainly because it contains a certain amount of lignin, which degrades relatively slowly. The short lag phase of PBAT in the first 21 days of the degradation process can be attributed to the dominance of hydrolysis in the initial stage of degradation. It takes a considerable amount of time for microorganisms to adhere to and settle on the material’s surface. The degradation rate increased steadily from 14 to 91 days and eventually reached 81.9%. The microorganisms adhering to the surface of the samples produced various types of depolymerization enzymes, such as lipase, Proteinase, and cellulase. These enzymes broke the ester bonds in the molecular chain of PBAT into small molecules or oligomers through hydrolysis and enzymatic reactions. The resulting compounds were then decomposed via microbial metabolism into CO_2_ and H_2_O. Compared to neat PBAT, the lag phase of PBAT/RF composites with 20% reed fibers was reduced by 23.8%, and the biodegradation rate increased by 11.8% after 91 days. This is consistent with the research results of Giri et al., which confirm that MCC enhances the degradation of PBAT-based composites [41]. 

**Figure 10:**
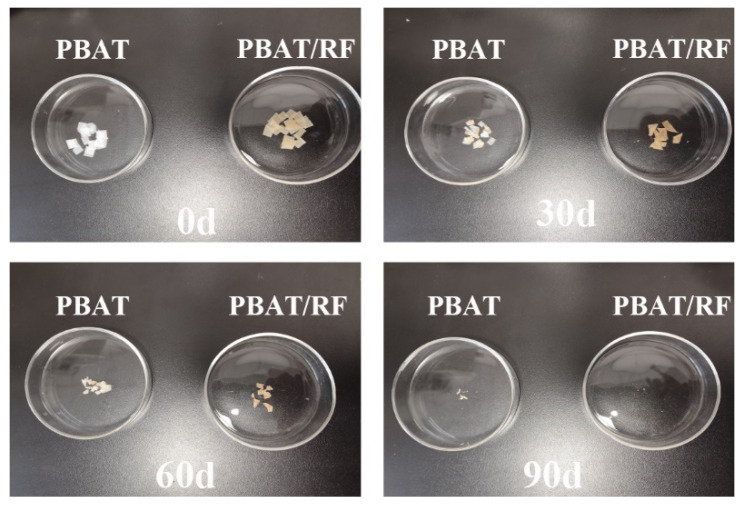
Photography of PBAT and PBAT/RF composites before and after composting degradation for 30, 60, and 90 days.

From Figure 10, it is evident that the initial samples of PBAT and PBAT/RF composites had smooth surfaces. However, after 30 days of degradation, the surfaces appeared yellowish-brown with dark spots. This may be caused by microbial colonies or residual compost. As degradation progressed, the surface of the samples became rough. Both PBAT and PBAT/RF composites became very brittle, and disintegration was visible. After 3 months of decomposition, the majority of the samples had disappeared, leaving behind only a minimal amount of tiny fragments measuring 1 to 2 mm. This effect was particularly noticeable in the PBAT/RF composites.

**Figure 11:**
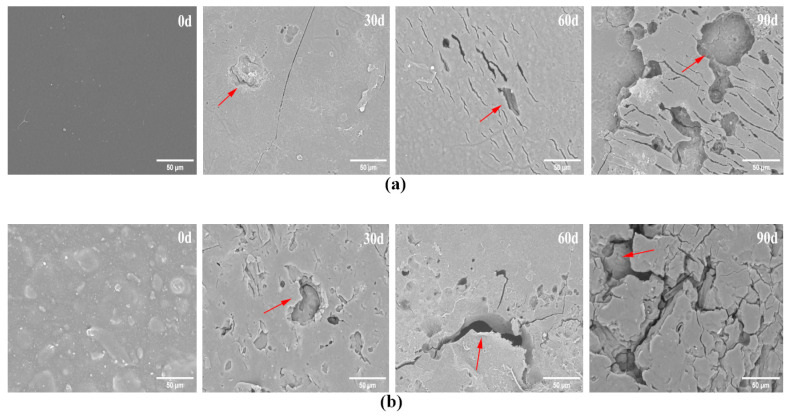
SEM micrographs of PBAT (**a**) and PBAT/RF composites (**b**) before and after composting degradation for 30, 60, and 90 days. (Red arrows indicate cracks and pores that form on the surface of PBAT and PBAT/RF composites after composting degradation).

Figure 11a,b show SEM images of PBAT and PBAT/RF composites during composting biodegradation. The PBAT film samples were observed to have eroded surfaces, along with fine cracks, on the 30th day of composting degradation. As the degradation time increased, the cracks became more pronounced, and pores started to appear. On the 90th day, large areas of necrosis were observed on the surface of PBAT, indicating severe microbial erosion. More pronounced signs of surface deterioration could be seen in the PBAT/RF composites at the same degradation time, indicating that they are more susceptible to degradation than neat PBAT. The hydrophilic groups in the reed fiber easily absorb water and microorganisms from the compost, which accelerates the hydrolysis and microbial consumption. This leads to the loss of reed fibers from the surface of the PBAT and the formation of cavities and cracks of various sizes. As a result, the area of the composites interacting with water and microorganisms increases, enhancing the biodegradability of the composites.

Compost degradation of PBAT/RF composites is a biochemical process that involves hydrolysis for non-biodegradation, and enzymatic degradation for biodegradation. Firstly, the material is exposed to water molecules in the composting environment, which leads to the breakdown of the molecular backbone and a reduction in molecular weight. Meanwhile, microorganisms proliferate and multiply on the surface of the material [42]. The composite material exhibits good hydrophilicity, which enhances hydrolysis and microbial attachment, thereby accelerating the process. Secondly, microorganisms secrete depolymerization enzymes (such as lipase, protease, etc.) and cellulose hydrolases outside the cell [43]. The extracellular depolymerization enzymes facilitate the hydrolysis of ester bonds and the degradation of PBAT, leading to surface erosion and the exposure of more reed fibers. The reed fibers serve as substrates for cellulase hydrolysis, breaking down into sugars that provide an excellent carbon source for microbial growth. This increase in the number of microorganisms around the material enhances the enzymatic degradation rate of the composite material. The microorganisms can increase the porosity of the composites by consuming the reed fibers, creating additional adhesion sites for the microorganisms, and enhancing the accessibility of the enzymes [44]. This process accelerates the degradation rate of the composites. Finally, oligomers and monomers produced by PBAT in the presence of depolymerases and other enzymes enter the microbial cell to be metabolized into small molecules for excretion outside the cell.

## 4. Conclusions

Reed grows extensively in China, and microfibrillated reed fiber can be used as natural fillers in PBAT composites. We investigated the enzymatic and composting degradation of PBAT/RF composites, and we found that the presence of reed fiber can enhance the degradation of PBAT. Regarding enzymatic degradability, lipase exhibited the most significant impact on the mass loss of PBAT/RF. Following a degradation period of 15 days, the mass loss of PBAT/RF was higher than that of PBAT. The findings of the study indicate a significant increase in water absorption and mass loss in the PBAT/RF composites compared to PBAT. This can be explained by the fact that the reed fibers enhance the hydrophilicity of the composites, and hydrolysis is a crucial rate-limiting step in the degradation of PBAT. The microstructures of the samples revealed that the surface of PBAT/RF exhibited more prominent pores and grooves. The DSC results showed that the melting temperatures and crystallinity of all the samples increased during the degradation process. However, the changes in PBAT/RF films were more significant than those in the neat PBAT films. The peaks at 2957 cm^−1^ and 1712 cm^−1^ in the FTIR spectrum weakened as the degradation time increased. This suggests that the macromolecular chains of PBAT and PBAT/RF composites were disrupted during the degradation process. These experimental results confirmed that the inclusion of reed fiber accelerated the degradation of PBAT. 

The results of the composting degradation also showed that the degradation rate of the PBAT/RF sample films was higher than that of pure PBAT during the test. The degradation rate of the PBAT/RF composite was 92.4 ± 2.4% after 3 months of composting, which was 11.8% higher than that of PBAT. The inclusion of reed fibers facilitated the penetration of moisture into the composite samples and potentially promoted microbial colonization. SEM images showed that much larger voids and cracks appeared on the surface of the PBAT/RF film, which resulted in faster and more efficient biodegradation compared to PBAT. 

This study provides a unique approach to evaluate reed resources. Reed fibers have the potential to serve as cost-effective fillers in a PBAT matrix, resulting in the production of environmentally friendly green composites that are biodegradable. The high biodegradability of this material makes it a viable and sustainable alternative to disposable plastics, particularly in the production of film products.

## Figures and Tables

**Table 1:** Properties of the controlled compost.

Properties	Value
Ph	7.20 ± 0.02
Total dry solids (%) ^a^	50.1 ± 0.13
Volatile solids (%) ^b^	18.6 ± 0.09
Total organic carbon content (%)	12.62 ± 0.07
Cabon/nitrogen ratio	14.9 ± 0.1

^a^ The total amount of dry solids is obtained by drying a known volume of compost at approximately 105 °C for 12 h. ^b^ The amount of volatile solids is determined by subtracting the residue of a known volume of compost after it has been incinerated at approximately 550 °C for 2 h.

**Table 2:** Thermal Properties of PBAT and PBAT/RF composites before and after various enzymatic degradation for 3, 9 and 15 days.

Samples	Time (Days)	*T*_*m*1_ (°C)	*T*_*m*2_ (°C)	Δ*H*_*m*_ (J/g)	*X*_*c*_ (%)	Samples	Time (Days)	*T*_*m*1_ (°C)	*T*_*m*2_ (°C)	Δ*H*_*m*_ (J/g)	*X*_*c*_ (%)
PBAT-LIP	0	-	126.5	21.57	18.92	PBAT/RF-Lip	0	-	125.5	20.25	22.20
	3	-	127.5	23.18	20.33		3	-	128.1	22.84	25.04
	9	124.1	128.5	24.82	21.77		9	123.5	129.8	23.66	25.94
	15	123.9	129.2	25.77	22.61		15	123.3	130.2	24.75	27.14
PBAT-CEL	0	-	126.1	20.66	18.12	PBAT/RF-Cel	0	-	125.8	20.98	23.00
	3	-	126.7	20.84	18.28		3	-	126.8	20.27	22.23
	9	-	127.3	21.67	19.01		9	-	127.2	22.15	24.29
	15	-	127.1	22.51	19.25		15	-	127.6	22.84	25.04
PBAT-PRO	0	-	125.5	21.53	18.89	PBAT/RF-Pro	0	-	126.6	22.07	24.20
	3	-	126.0	21.89	19.20		3	-	127.2	23.17	25.41
	9	-	126.2	22.17	19.45		9	-	128.8	24.06	26.38
	15	-	127.8	24.67	21.64		15	-	129.5	25.59	28.06
PBAT-EET	0	-	126.2	21.75	19.08	PBAT/RF-Est	0	-	126.4	22.65	24.84
	3	-	126.8	22.44	19.68		3	-	128.0	23.29	25.54
	9	-	126.8	23.66	20.75		9	-	128.1	24.95	27.36
	15	-	128.3	24.59	21.57		15	-	129.1	25.81	28.30

## Data Availability

Data are contained within the article.
